# Bioactive granular hydrogels for infection control and immune microenvironment remodeling in wound regeneration

**DOI:** 10.1093/rb/rbag158

**Published:** 2026-07-29

**Authors:** Yuyan Hua, Yung-Chiang Liu, Zhili Qiu, Yin Liu, Guoyan Li, Zhilin Wei, Yuefang Shen, Huiyuan Chen, Bihui Ren, Enhui Zhou, Jinfeng Wang, Duoduo Zhang, Peng-Yuan Wang

**Affiliations:** Oujiang Laboratory, Key Laboratory of Alzheimer’s Disease of Zhejiang Province, Institute of Aging, Wenzhou Medical University, Wenzhou, Zhejiang 325000, China; Oujiang Laboratory, Key Laboratory of Alzheimer’s Disease of Zhejiang Province, Institute of Aging, Wenzhou Medical University, Wenzhou, Zhejiang 325000, China; Oujiang Laboratory, Key Laboratory of Alzheimer’s Disease of Zhejiang Province, Institute of Aging, Wenzhou Medical University, Wenzhou, Zhejiang 325000, China; Oujiang Laboratory, Key Laboratory of Alzheimer’s Disease of Zhejiang Province, Institute of Aging, Wenzhou Medical University, Wenzhou, Zhejiang 325000, China; Oujiang Laboratory, Key Laboratory of Alzheimer’s Disease of Zhejiang Province, Institute of Aging, Wenzhou Medical University, Wenzhou, Zhejiang 325000, China; Oujiang Laboratory, Key Laboratory of Alzheimer’s Disease of Zhejiang Province, Institute of Aging, Wenzhou Medical University, Wenzhou, Zhejiang 325000, China; Oujiang Laboratory, Key Laboratory of Alzheimer’s Disease of Zhejiang Province, Institute of Aging, Wenzhou Medical University, Wenzhou, Zhejiang 325000, China; Oujiang Laboratory, Key Laboratory of Alzheimer’s Disease of Zhejiang Province, Institute of Aging, Wenzhou Medical University, Wenzhou, Zhejiang 325000, China; Oujiang Laboratory, Key Laboratory of Alzheimer’s Disease of Zhejiang Province, Institute of Aging, Wenzhou Medical University, Wenzhou, Zhejiang 325000, China; Oujiang Laboratory, Key Laboratory of Alzheimer’s Disease of Zhejiang Province, Institute of Aging, Wenzhou Medical University, Wenzhou, Zhejiang 325000, China; Oujiang Laboratory, Key Laboratory of Alzheimer’s Disease of Zhejiang Province, Institute of Aging, Wenzhou Medical University, Wenzhou, Zhejiang 325000, China; Oujiang Laboratory, Key Laboratory of Alzheimer’s Disease of Zhejiang Province, Institute of Aging, Wenzhou Medical University, Wenzhou, Zhejiang 325000, China; Oujiang Laboratory, Key Laboratory of Alzheimer’s Disease of Zhejiang Province, Institute of Aging, Wenzhou Medical University, Wenzhou, Zhejiang 325000, China

**Keywords:** granular hydrogel, antibacterial, antioxidant, immunomodulation, wound healing

## Abstract

Bacterial infection remains a major barrier to effective wound healing by disrupting immune homeostasis, sustaining chronic inflammation and impairing tissue regeneration. Herein, we present a green, sustainable strategy for fabricating antibacterial, immunomodulatory bioactive granular hydrogels (GHs) for infected wound regeneration. An amino-alcohol ether prepolymer (MP) was first synthesized via epoxy–amine click chemistry and subsequently complexed with the natural polyphenol tannic acid (TA), thereby triggering phase-separation-driven supramolecular self-assembly into GHs without additional crosslinkers. To elucidate the polymer assembly mechanism and identify the bioactive concentration threshold, agarose was introduced as a fourth component to construct A/MP@TA GHs. The results showed that increasing the agarose content progressively transformed the granular architecture into a sheet-like network, whereas A/MP@TA_3_, which represents the lowest agarose ratio that preserves the granular morphology, exhibited potent antibacterial and antioxidant activities and enhanced fibroblast migration. In a bacteria-infected wound, A/MP@TA_3_ still markedly accelerated wound closure while promoting collagen deposition and angiogenesis. Mechanistically, sustained TA release reprogrammed the microenvironment by activating the KEAP1/Nrf2/HO-1 and suppressing NF-κB signaling, thereby driving macrophage polarization toward a pro-regenerative M2 phenotype. This work establishes a simple, cost-effective and environmentally friendly platform for fabricating multifunctional hydrogel dressings and provides a biomaterial-based strategy for remodeling the immune microenvironment.

## Graphical abstract

**Graphical Abstract:**
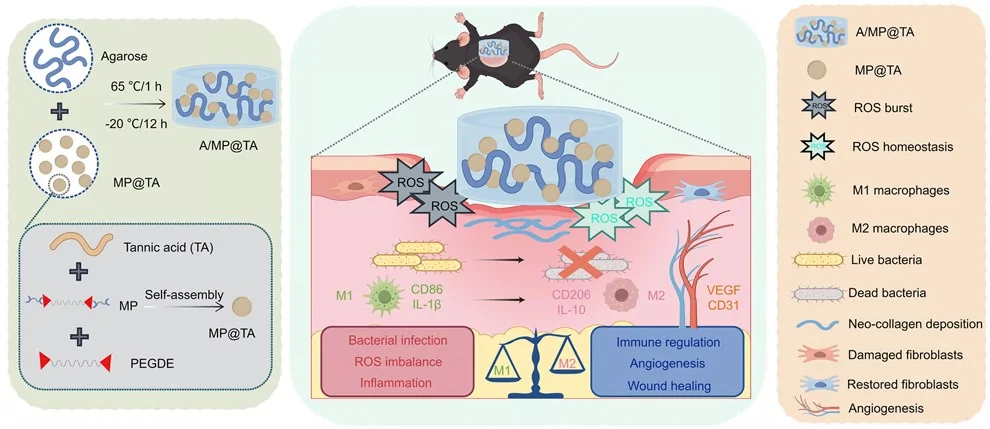

## Introduction

Infected wounds, particularly those complicated by drug-resistant bacterial infections, remain a major clinical challenge in regenerative medicine [1]. These wounds are characterized by persistent inflammation, excessive accumulation of reactive oxygen species (ROS) and bacterial biofilm formation, collectively disrupting tissue regeneration and prolonging the inflammatory phase of healing. The increasing prevalence of antibiotic-resistant pathogens, such as *Methicillin-resistant Staphylococcus aureus* (MRSA), further compromises the effectiveness of conventional antimicrobial therapies. Consequently, biomaterials, leveraging their intrinsic physical and chemical properties [2, 3], can simultaneously address bacterial infection, oxidative stress and immune dysregulation and have attracted considerable attention as advanced therapeutic strategies for managing infected wounds [4].

Granular hydrogels (GHs), particularly microporous annealed particle (MAP) hydrogels assembled from microgel building blocks, have recently emerged as promising materials for tissue repair [5, 6]. Unlike conventional homogeneous hydrogels, GHs form an interconnected porous architecture by packing microgel particles. This intrinsic microporosity enables rapid cell infiltration and efficient mass transport without requiring prior material degradation, thereby supporting vascularization and tissue remodeling. In addition, the large interfacial area of microgel particles provides abundant binding sites for incorporating and locally delivering bioactive molecules, making GHs attractive platforms for therapeutic modulation of complex wound microenvironments [7, 8]. However, most current approaches to functionalizing GHs rely on multicomponent hybrid systems or complex chemical modifications to introduce antibacterial or antioxidant functions, thereby increasing fabrication complexity and potentially hindering scalable, environmentally sustainable production.

A fully aqueous, green fabrication strategy to construct GHs with tunable bioactivity was previously developed in our group [9]. In this system, a poly(amino alcohol ether) (MP) prepolymer is first synthesized via a nucleophilic ring-opening click reaction between epoxides and amines. GHs are subsequently formed via a simple one-pot process by mixing MP with hydroxyl-rich molecules, such as polyphenols, which triggers complexation-mediated phase separation and self-assembly into granular structures. The morphology and size of the resulting granules can be readily tuned by adjusting the component ratios. Notably, polyphenols not only actively participate in GH formation through their abundant hydroxyl groups but also contribute intrinsic biological activities.

Natural polyphenols have recently emerged as versatile building blocks for functional biomaterials [10]. Owing to their rich phenolic hydroxyl groups, polyphenols can mediate supramolecular assembly through hydrogen bonding, hydrophobic interactions and dynamic coordination, while simultaneously providing antibacterial, antioxidant and anti-inflammatory functions. Among them, tannic acid (TA), a plant-derived polyphenol, has been widely reported to disrupt bacterial membranes, inhibit biofilm formation, scavenge ROS and regulate macrophage polarization, thereby facilitating the restoration of a pro-healing immune microenvironment [11]. TA is a naturally occurring polyphenol characterized by a glucose core esterified with multiple galloyl groups, most commonly represented by the molecular formula C_76_H_52_O_46_. Its hydrophilicity arises from the abundance of hydroxyl (-OH) groups, while the aromatic galloyl rings and the central glucose scaffold contribute hydrophobic domains. This unique combination of hydrophilic and hydrophobic moieties endows TA with pronounced amphiphilic properties [12]. Furthermore, TA has been shown to promote wound healing by modulating fibroblast migration and proliferation, thereby facilitating tissue regeneration and remodeling [13]. Simultaneously, TA plays a crucial role in reshaping the wound microenvironment by inhibiting pro-inflammatory factors (e.g. by suppressing activation of the NF-κB signaling pathway) and orchestrating the macrophage subtypes from M1-enriched (pro-inflammatory) to M2-enriched (pro-repair/anti-inflammatory) microenvironment [14–16]. These cell-level regulatory mechanisms underscore TA’s potential as a key functional component in the treatment of infected wounds.

Previously, bioactive TA-loaded GHs were fabricated for diabetic wound healing, leveraging TA’s amphiphilic nature, which facilitates phase separation upon mixing with the MP prepolymer and PEGDE [9]. Specifically, TA interacted with the polymer network via hydrogen bonding, electrostatic interactions and hydrophobic π-π stacking, thereby forming stable polymer-polyphenol complexes. These multivalent interactions promoted phase separation and ultimately drove the self-assembly of the particulate architecture.

Building on this concept, the present study further explores the structural tunability of polyphenol-mediated GHs by introducing a fourth component. Specifically, agarose (a hydroxyl-rich yet biologically inert polysaccharide hydrogel) was incorporated into the GH system to investigate whether additional hydroxyl-containing polymers could participate in and regulate granular assembly. This design provides a controlled framework for examining how cooperative or competitive interactions between natural polyphenols and polymeric networks influence GH formation and functionality. Using this strategy, we further determined the minimal TA concentration required to initiate granular structure formation while retaining bioactivity. Remarkably, even with minimal TA incorporated within the granular structure, the resulting GH maintained pronounced antioxidant, antibacterial and anti-inflammatory activities. These findings highlight the functional efficiency of this design and provide new insights into polyphenol-mediated GH systems for antibacterial wound dressings.

## Materials and methods

### Materials

TA (Mn ≈ 1701.2Da), 2,2-Diphenyl-1-picrylhydrazyl (DPPH), agarose (Aga, *M*n ≈ 12000 kDa) and poly(ethylene glycol) diglycidyl ether (PEGDE, *M*n ≈ 3400 Da) were purchased from Sigma-Aldrich (St. Louis, MO, USA). 2-Methyl-1,5-diaminopentane (MD, *M*n ≈ 116.21 Da) was obtained from Tixiai (Shanghai) Chemical Industry Development Co., Ltd. Nile red and Folin reagent were sourced from Beijing Solarbio Science & Technology Co. Ltd. (Beijing, China). Fluorescein isothiocyanate isomer I (FITC, 90%) was obtained from YEASEN. The Total Antioxidant Capacity Assay Kit (FRAP method) was purchased from Shanghai Beyotime Biotechnology Co., Ltd (Shanghai, China). The commercial absorbable gelatin hemostatic sponge was purchased from Nanchang Hushida Medical Technology Co., Ltd. (HSD-B), China.

### Preparation of A/MP@TA GHs

A fully aqueous, green fabrication method was used to prepare the polyphenol-based GHs, as described in our previous study [9]. First, 0.29 g of MD was dissolved in 7.47 g of ultrapure water, then 0.83 g of PEGDE was added, and the mixture was thoroughly mixed. The mixture was reacted at 65°C (water bath) for 30 min to generate the poly(amino alcohol ether) MP prepolymer, then cooled down (water bath) for 5 min. Next, an 8 wt% TA solution was added to the MP prepolymer at a volume ratio of 2:1 (6 mL MP: 3 mL TA). Additionally, 1.2 g of PEGDE was added to the 9 mL MP@TA solution. Finally, a 2 wt% aqueous agarose solution at various volume ratios was added to the MP@TA solution to form A/MP@TA gels. The A/MP@TA gel was reacted at 65°C (water bath) for 1 h. Then, the A/MP@TA GHs were frozen at −20°C for 24 h to consolidate the internal structures. The resulting A/MP@TA hydrogel was immersed in ultrapure water for 24 h.

The volume ratios of agarose-to-MP@TA were designed as 5:1, 4:2, 3:3, 2:4, 1:5 and 0.5:5.5, resulting in six GHs which were A/MP@TA_1_, A/MP@TA_2_, A/MP@TA_3_, A/MP@TA_4_, A/MP@TA_5_ and A/MP@TA_6_, respectively.

The above reaction conditions were selected to ensure an optimal reaction. MP prepolymer does not form a gel at various temperatures (i.e. 45°C, 55°C, 65°C and 75°C). Therefore, a 65°C, 30 min condition was selected to partially cross-link the MP prepolymer. In the second step, adding TA and PEGDE to the MP prepolymer resulted in MP@TA GHs that gradually formed gels after 30 min (Supplementary Table S1). To ensure a complete reaction, 65°C for 1 h was selected to form stable MP@TA GHs. In this study, additional agarose shortens the gelation time to within 10 min.

### Characterization of GHs

#### Scanning electron microscopy

The hydrogel microstructure was characterized using field emission scanning electron microscopy (FE-SEM; SU8010, HITACHI, Japan). The samples were fully dehydrated by vacuum freeze-drying, followed by 90 seconds of platinum sputtering to enhance surface conductivity. Subsequently, the images was captured under 5.0 KV.

#### Fourier transform infrared spectroscopy

The chemical structures of the A/MP@TA GH were characterized using Fourier transform infrared spectroscopy (FTIR, Nicolet iS50, Thermo Scientific Instrument, USA). Spectral acquisition was performed in attenuated total reflection (ATR) mode with the following parameters: a wavenumber range of 4000–500 cm^−1^, a resolution of 4 cm^−1^ and 32 cumulative scans to enhance the signal-to-noise ratio. Each group of samples was tested in triplicate for independent replication.

#### Mechanical property

The rheological behavior of the hydrogel was characterized using a hybrid rheometer (TA Instruments, USA). Fully hydrated cylindrical samples (diameter 20 mm, height 4 mm) were immersed in deionized water for 24 h prior to testing, followed by oscillatory frequency sweep measurements. The tests were conducted under a constant dynamic strain amplitude of 0.5%, with an angular frequency range of 0.1–100 rad/s. Each sample group was tested in triplicate for independent replication.

#### Swelling behavior

The swelling behavior was tested in PBS buffer at room temperature. Freeze-dried samples of known initial weight (*W_0_*) were immersed in PBS buffer and removed at predetermined time points (1, 2, 5, 10, 20, 40 min; 1, 2, 4, 6, 8, 12, 24 h). After removing surface liquid with filter paper, the weight (*W_t_*) was measured. The swelling ratio (SR) was calculated using the following formula:


$$\text{SR}\;(\%)=\left[\left(W_{t}-W_{0}\right)/W_{0}\right]\times 100\%,$$

where *W*_0_ and *W_t_* represent the initial weight of the freeze-dried sample before swelling and the weight after swelling for time *t*, respectively.

#### Drug release assay

To analyze the release profile of TA from the hydrogel, the Folin–Ciocalteu method was used to quantify the released amount. A 50 mg hydrogel sample was immersed in 5 mL of PBS buffer and incubated in a 37°C water bath. The release medium was collected at predetermined time points (1, 2, 4, 6, 8, 12, 24, 48, 72, 120 and 168 h). The concentration of TA was quantified, and the cumulative release amount was calculated accordingly.

### Antioxidant properties of the GHs

The antioxidant capacity of the hydrogel was evaluated using the total antioxidant capacity assay (FRAP method, Kit S0116, Beyotime) and DPPH radical-scavenging experiments. All experiments were performed independently in triplicate. A brief description of the DPPH radical-scavenging experiment is as follows: DPPH was dissolved in methanol to prepare a stock solution (0.05 mg/mL), which was then diluted with ultrapure water at a 3:1 ratio to prepare the working solution. An appropriate amount of the hydrogel sample was incubated with the working solution at room temperature for 10 min. A mixture of equal volumes of working solution and ultrapure water served as the blank control. After the reaction, the absorbance was measured at 517 nm. The DPPH scavenging activity was calculated using the following formula:


$$\mathrm{DPPH}\;\mathrm{scavenging}\;(\%)=[(A_{w}-A_{h})/A_{w}]\times 100\%,$$

where *A_w_* and *A_h_* represent the absorbance values at 517 nm for the blank control and hydrogel groups, respectively.

### Hemolytic assay

To evaluate the hemocompatibility of the hydrogel, the hemolysis rate was determined through a hemolysis assay. First, a 10 mg/mL solution was prepared by dissolving 10 mg of freeze-dried hydrogel in 1 mL of PBS. Then, 1 mL of this solution was mixed with 300 μL of anticoagulant acid citrate dextrose and fresh rabbit blood, and the mixture was incubated in a constant-temperature incubator at 37°C and 5% CO_2_ for 1 h. Deionized water and PBS-treated blood served as positive and negative controls, respectively. After incubation, all samples were centrifuged at 750 × *g* for 5 min, and the supernatant was collected. The absorbance (OD) was measured at 540 nm using a multimode microplate reader (VARIOSKAN LUX, Thermo Scientific, USA). The hemolysis rate was calculated using the following formula:


$$\mathrm{HR}\;(\%)=[({\mathrm{OD}}_{\mathrm{test}}-{\mathrm{OD}}_{\mathrm{pbs}})/({\mathrm{OD}}_{\mathrm{water}}-{\mathrm{OD}}_{\mathrm{pbs}})]\times 100\%,$$

where OD_test_, OD_water_ and OD_pbs_ represent the absorbance values of the experimental group, the positive control group (water) and the negative control group (PBS), respectively.

### Cytotoxicity assay

To evaluate the cytocompatibility of the hydrogel, NIH3T3 fibroblasts and RAW264.7 macrophages were seeded at densities of 1 × 10^4^ cells/well and 5 × 10³ cells/well, respectively, in 24-well and 96-well plates. After cell attachment, the medium was replaced with fresh medium supplemented with hydrogel extracts to continue culture. Cell viability and proliferation were assessed using live/dead staining and the CCK-8 assay after 24, 48 and 72 h of co-culture.

For live/dead staining, cells were stained with a kit (Calcein-AM/PI, C2015M, Beyotime), and images were captured using a confocal laser scanning microscope (CLSM, FV3000, Olympus, Japan). Cell proliferation was assessed using a CCK-8 kit (k1018, APExBIO, USA). Specifically, 10 μL of CCK-8 working solution was added to each well, followed by incubation. Absorbance was then measured at 450 nm using a multimode microplate reader (INFINITE 200 PRO, TECAN, Switzerland), and relative cell viability was calculated based on the absorbance values.

### Antioxidant activity for cell protection

To evaluate the intracellular antioxidant capacity of the A/MP@TA_3_ GH, NIH3T3 fibroblasts were seeded in 6-well plates at a density of 3 × 10^5^ cells per well. After cell attachment, the medium was replaced with fresh medium containing 100 μM H_2_O_2_ and hydrogel extracts, and the cells were incubated for an additional 24 h. Subsequently, cell viability was assessed using the CCK-8 assay. Meanwhile, intracellular ROS levels were examined by staining with the fluorescent probe DCFH-DA (S1105M, Beyotime, China). The ROS levels were observed using a CLSM (FV3000, Olympus, Japan), and the fluorescence intensity was quantitatively analyzed using a flow cytometer (CytoFlex LX, Beckman Coulter, USA).

### Cell migration assays

In addition, a scratch cell migration assay was conducted to assess the hydrogel’s effect on cell migration under oxidative stress conditions. When the confluence of NIH3T3 cells exceeded 90%, scratches were created in the monolayer using a 1-mL sterile pipette tip. The cells were then cultured in medium containing 100 μM H_2_O_2_ and hydrogel extracts. Images were captured at 0, 12, 24 and 48 h using an inverted microscope (EVOS™ XL Core, Thermo Scientific, USA), and the closure rate of the scratch area was quantified in ImageJ to evaluate cell migration.

### Antimicrobial assay

The antibacterial effects of the hydrogel against MRSA (25923, ATCC) and *Escherichia coli* (*E. coli*, 8739, ATCC) were evaluated using colony counting and the inhibition zone method. For the colony-counting assay, bacteria were cultured in a medium containing the hydrogel’s release products. Specifically, bacterial suspensions (5 × 10^6^ CFU/mL) were mixed with PBS at a 1:6 ratio in 24-well plates to a final volume of 2 mL, and the plates were co-cultured at 37°C for 12 h. Subsequently, 100 µL of the co-culture mixture was spread onto LB agar plates, incubated at 37°C for 24 h, and colony counts were performed to assess the hydrogel’s impact on bacterial viability. For the inhibition zone assay, bacteria were cultured to the logarithmic phase and adjusted to a concentration of 1 × 10^7^ CFU/mL with PBS. Sterilized hydrogel disks with a diameter of 6 mm were placed on LB agar plates that had been pre-spread with 100 µL of bacterial suspension. After incubation at 37°C for 24 h, the diameter of the inhibition zones was measured. All experiments were performed in triplicate.

### Molecular docking analysis

To investigate the potential protein targets of gallic acid, molecular docking simulations were performed using the CB-Dock2 online server (http://183.56.231.194:8001/cb-dock2/php/index.php). This platform integrates cavity detection, blind docking and homology template-fitting functions. The three-dimensional structures of human Heme Oxygenase-1 (HO-1, PDB: 3HOK), NF-κB (p65, PDB: 1BFT), Kelch-like ECH-associated protein 1 (KEAP1, PDB: 4L7B) and Peroxisome Proliferator-activated Receptor γ (PPARγ, PDB: 2PRG) were obtained from the Research Collaboratory for Structural Bioinformatics (RCSB) Protein Data Bank (PDB) without additional modifications. Gallic acid (CID: 370) was retrieved from the PubChem database and used as the ligand. Docking was performed with default parameters: search exhaustiveness = 20, energy range = 5.0 kcal/mol and maximum number of binding conformations = 20. The largest predicted cavity in each protein was selected as the binding site. The final docking conformations were ranked by Vina score, and the optimal conformation was selected for interaction analysis to identify hydrogen bonds and electrostatic interactions. All docking results are presented as predicted binding affinities.

### Western blot

THP‑1 cells were cultured in 6‑well plates. After adherence, the cells were stimulated with LPS for 24 h, then incubated with the hydrogel leachate for an additional 48 h. The cells were then lysed in RIPA buffer (Beyotime, P0013B) supplemented with protease and phosphatase inhibitors. The lysates were centrifuged at 12 000 rpm for 30 min at 4°C, and the supernatants were collected. Protein concentrations were determined using a BCA protein assay kit. A total of 30 μg of protein from each sample was loaded onto SDS‑PAGE gels for electrophoresis and subsequently transferred onto PVDF membranes. The membranes were incubated overnight at 4°C with specific primary antibodies against iNOS (18985‑1‑AP, Proteintech), CD206 (ab64693, Abcam), KEAP1 (61257, Genuin Biotech), Nuclear Factor Erythroid 2-Related Factor 2 (Nrf2, T55136, Abmart), p‑NFκB (AF3387, Affinity), NF-κB (AF5006, Affinity), HO‑1 (10701‑1‑AP, Proteintech), PPARγ (16643‑1‑AP, Proteintech) and GAPDH (10494‑1‑AP, Proteintech). Afterward, the membranes were incubated with the corresponding secondary antibodies: HRP-conjugated goat anti-mouse IgG H + L (SA00001‑1, Proteintech) and HRP-conjugated goat anti-rabbit IgG H + L (SA00001‑2, Proteintech) for 2 h at room temperature. Protein bands were visualized using an ECL detection kit (Thermo Fisher Scientific). GAPDH was used as a loading control.

### *In vivo* infected wound healing assay

All animal procedures performed in this study were in strict accordance with the guidelines of the National Institutes of Health Guide for the Care and Use of Laboratory Animals. The protocol was approved by the Institutional Animal Care and Use Committee (IACUC) of Oujiang Laboratory (approval number: OJLAB26010601). All surgeries were performed under deep anesthesia, and every effort was made to minimize suffering.

To avoid interference from fighting among male mice over wounds, this study used 8-week-old female C57BL/6 mice (weight: 25–30 g) to establish an MRSA-infected skin wound model. Under anesthesia with 2.5% isoflurane gas, two circular full-thickness skin wounds with a diameter of 8 mm were created on the back of each mouse. The wounds were inoculated with 10 µL of MRSA suspension (1 × 10^8^ CFU/mL) and infected for 3 days.

Subsequently, the mice were randomly divided into four groups: PBS control group, hemostatic sponge group, TA solution group and hydrogel treatment group. Corresponding materials were applied to the wounds. During the treatment period, the dressings in all four groups were replaced on Days 3 and 7, and wound photographs were taken on Days 3, 7 and 14. On Day 14 after the intervention, all animals were euthanized. Skin tissue from the wound site and major organs (heart, liver, spleen, lung and kidney) were collected and fixed in 4% paraformaldehyde. Subsequent paraffin embedding and sectioning were performed for further analysis.

### Immunomodulation and tissue regeneration assays

After dewaxing, the paraffin sections underwent antigen retrieval and blocking. The sections were then incubated overnight at 4°C with specific primary antibodies: CD86 (ab220188, abcam), CD206 (ab64693, abcam), IL-1β (ab283818, abcam), IL-10 (ARC9102, Thermo Fisher Scientific), vascular endothelial growth factor (VEGF, ab32152, abcam), CD31 (ab182981, abcam) and Ki67 (9449 T, CST). The next day, the sections were incubated at room temperature with corresponding secondary antibodies: Alexa Fluor® 488-conjugated goat anti-mouse IgG H + L (ab150117, abcam) and Alexa Fluor® 555-conjugated goat anti-rabbit IgG H + L (ab150078, abcam), followed by nuclear staining with DAPI (708939ES03, YEASEN). After mounting, whole-slide images were acquired using an automated digital slide scanner (Axioscan 7, ZEISS). Finally, fluorescence signals were quantified using ImageJ.

### Statistical analysis

The differences between the experimental groups were analyzed using the t-test to assess statistical significance. Data are presented as the mean ± standard deviation (SD) from three independent experiments. The statistical analyses were performed using a one-way ANOVA test in GraphPad Prism (version 9.0, GraphPad Software, Inc., CA, USA). The difference thresholds were set at *P < 0.05; **P < 0.01; ***P < 0.001.

## Results and discussion

### Fabrication and characterization of A/MP@TA GHs

**Figure 1:**
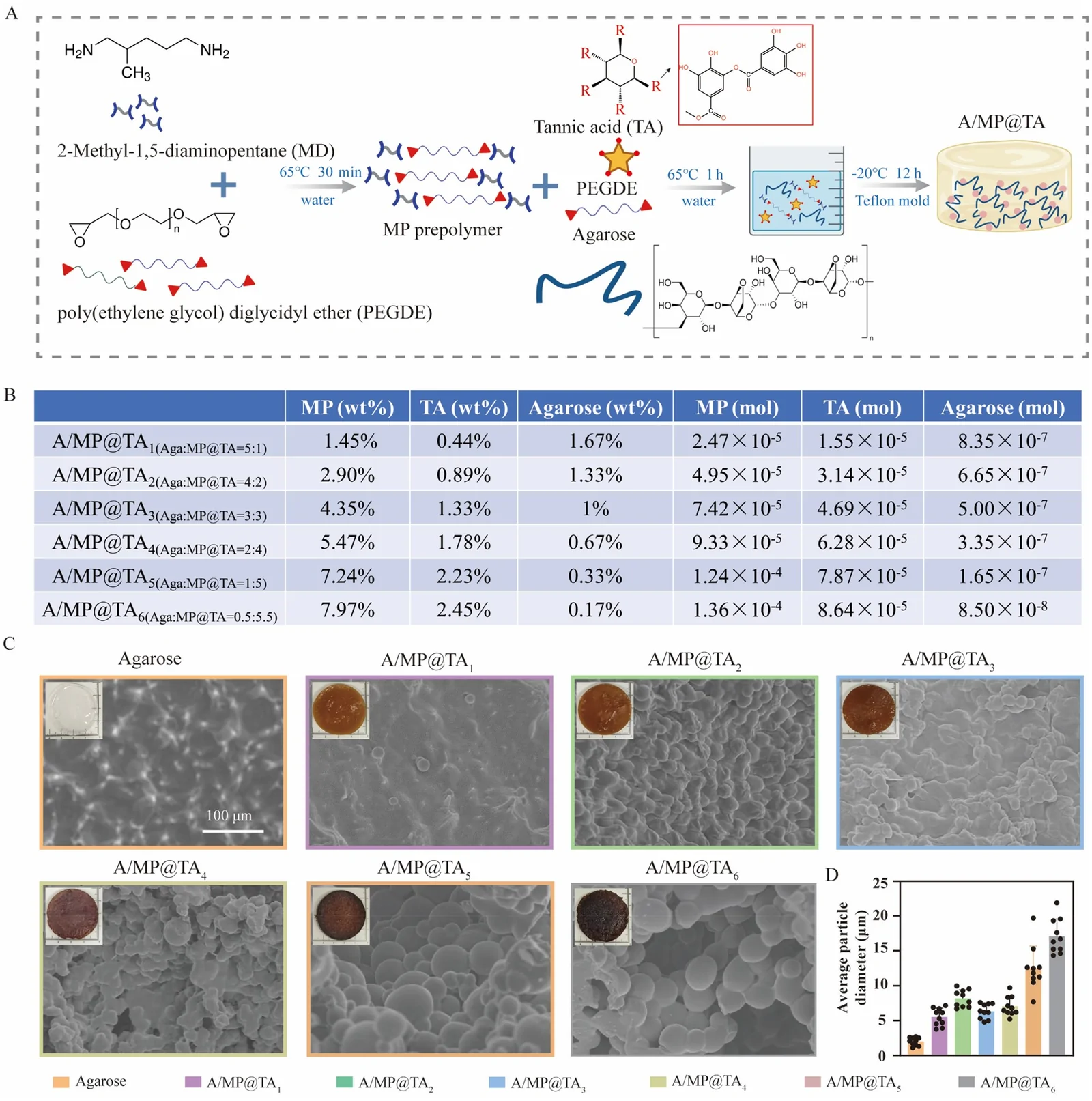
Synthesis and structural characterization of A/MP@TA hydrogels. (**A**) Schematic illustration of the synthetic pathway. The process involves pre-polymerizing MD and poly(ethylene glycol) diglycidyl ether (PEGDE), followed by incorporating TA and agarose to form the final GH. (**B**) Compositional ratios of the hydrogel systems, detailed weight percentages (wt%) and molar amounts (mol) for all A/MP@TA GHs. (**C**) SEM images of pure agarose and A/MP@TA GHs with varying TA concentrations (scale bars: 100 μm). (**D**) Statistical analysis of average particle diameters of all A/MP@TA GHs. Data are presented as mean ± SD (*n* = 10).

A new four-component granular hydrogel (A/MP@TA) was fabricated via a rapid, green strategy that integrates the epoxy-amine click reaction, complexation-induced phase separation and cryogelation, as reported in our previous study [9]. Briefly, MD reacted with PEGDE to generate a poly(amino alcohol ether) prepolymer (MP) rich in amine groups, which subsequently engaged in strong hydrogen-bonding and ionic interactions with the phenolic hydroxyl groups of TA. These interactions drove molecular complexation, while the hydrophobic aromatic rings of TA further induced phase separation and supramolecular assembly (Figure 1). The mixture of MP prepolymer, PEGDE and TA (MP@TA GHs) gradually formed gels after 30 min and fully reacted at 65°C for 1 h, followed by freezing (−20°C) to form stable GHs. Without TA, MP prepolymer and PEGDE can rapidly form a gel within 10 min but do not form a granular structure, indicating TA’s critical role in GH formation. Agarose can form a gel quickly at room temperature; therefore, after the introduction of agarose, the A/MP@TA gel formed within 10 min. Rapid agarose gelation disrupts TA-mediated granular structure formation (Figure 1A and Supplementary Table S1).

SEM analysis confirmed the presence of interconnected porous networks with granular structures at low agarose content in A/MP@TA_4-6_ (Figure 1B). Increasing the agarose ratio progressively reduced particle size and density, and at A/MP@TA_1-3_, the structure was dominated by agarose lamellae with small MP@TA particles attached to the matrix (Figure 1C). The granular size also decreased as the MP@TA ratio decreased (Figure 1D). These results indicate that MP@TA forms the primary particulate domains, and TA contributes to particle stabilization and functional activity. To evaluate the bioactivities at a low TA ratio, this study primarily tested the higher-agarose-content GHs, i.e. A/MP@TA_1-3_.

According to our and other studies, the phenolic hydroxyl groups and aromatic rings in TA enable TA to simultaneously form hydrophilic hydrogen bonds and hydrophobic aggregates, which are crucial for the formation of stable granular structures and condensed-phase droplets [9, 17, 18]. The phenolic hydroxyl groups in TA form complexes with the amino groups in the prepolymer, while the aromatic rings promote hydrophobic aggregation, thereby leading to liquid–liquid phase separation. Both properties are indispensable. Agarose is a nontoxic polysaccharide with distinct properties from TA (Supplementary Table S1); therefore, it is a good candidate for studying GH formation.

MP@TA GHs have previously shown excellent antibacterial and anti-inflammatory properties, thereby improving wound healing and management [9]. In this study, agarose was intentionally incorporated into the MP@TA GH system to alter the granular structure, which allows us to explore the self-assembly process of A/MP@TA GH formation and determine the lower limit of bioactivity. The results suggested that A/MP@TA_1-3_ may exhibit limited bioactivity in antibacterial, antioxidant, anti-inflammatory and wound-healing effects.

**Figure 2:**
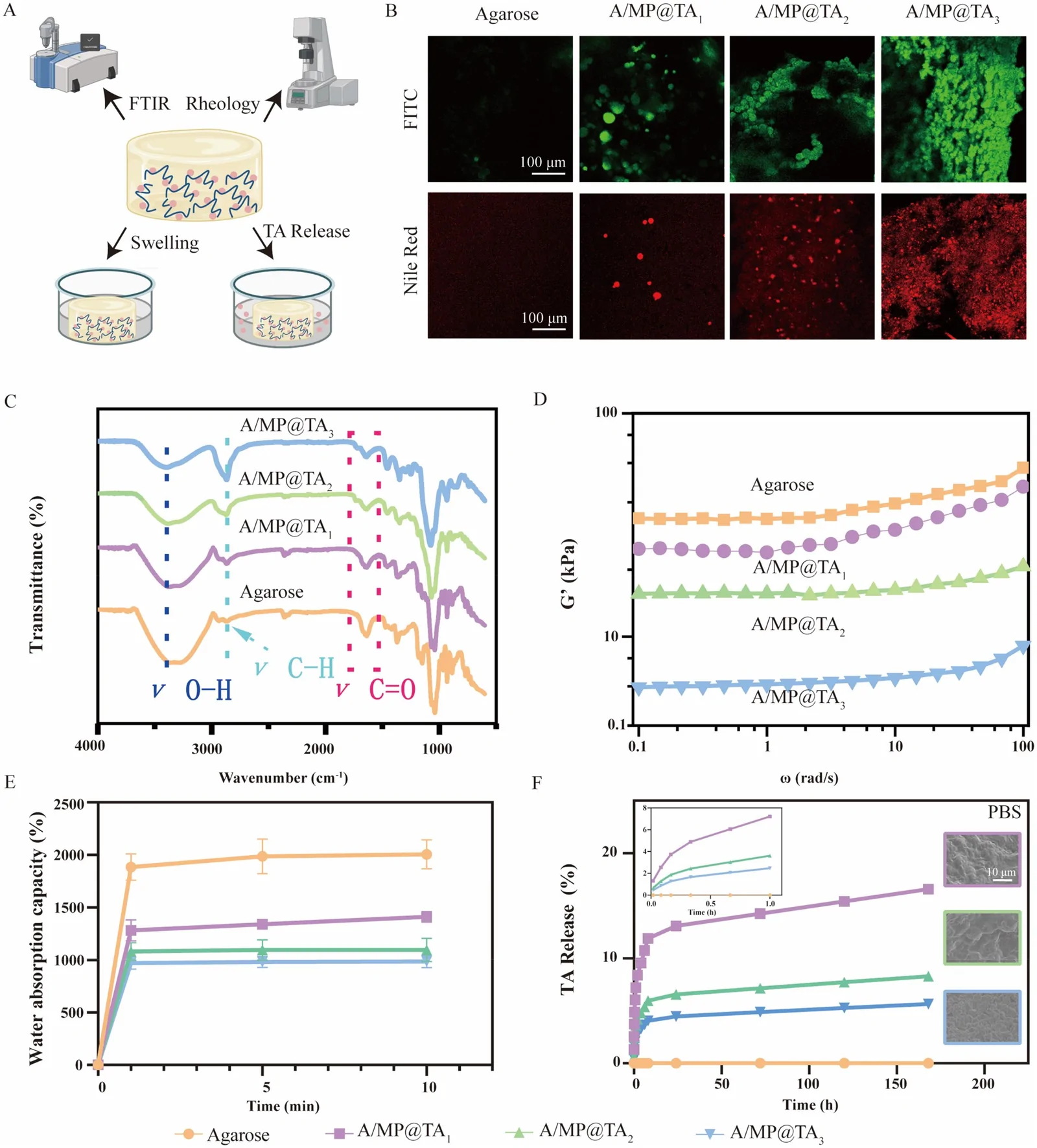
Physicochemical characterization and *in vitro* drug release kinetics of A/MP@TA hydrogels. (**A**) Schematic illustration of hydrogel characterization. Overview of the physicochemical and functional analyses performed on the A/MP@TA hydrogels, including ATR-FTIR spectroscopy, dynamic rheological testing, swelling behavior analysis and *in vitro* TA release studies. (**B**) Fluorescence labeling and distribution. Representative confocal fluorescence microscopy images of pure agarose and A/MP@TA hydrogels. Amine groups within the network are labeled with FITC (green), while hydrophobic domains are stained with Nile Red (red). Fluorescence images reveal the distribution of particles (scale bars: 100 μm). (**C**) ATR-FTIR spectra. Characterization of functional groups for agarose and various A/MP@TA formulations. Key vibrational modes are highlighted, including hydroxyl groups (νO-H), alkyl chains (νC-H) and carbonyl groups (νC = O). (**D**) Dynamic rheological properties. G’ as a function of frequency for the different hydrogel groups, illustrating the impact of MP and TA integration on the viscoelastic network strength. (**E**) Water absorption study. Time-dependent water uptake ratios of the hydrogels in aqueous media over 10 min. Data are presented as mean ± SD (*n* = 3). (**F**) *In vitro* cumulative release profile of TA. Sustained release amount of TA from the agarose, A/MP@TA_1_, A/MP@TA_2_ and A/MP@TA_3_ in PBS. The hydrogel system was monitored over 168 h (7 days). SEM micrographs represent the various GHs in PBS for 1 month.

The A/MP@TA_1-3_ GHs were further characterized (Figure 2A). Fluorescence labeling with FITC (an amine probe) and Nile red (a hydrophobic probe) further verified the formation of MP@TA particles (Figure 2B). Increasing MP@TA content enhanced FITC fluorescence intensity and expanded Nile red signals, indicating higher amine density and hydrophobic microdomains within the hydrogel network (Figure 2B). This property suggests potential for hydrophobic drug loading.

ATR-FTIR spectra revealed strong intermolecular interactions among the hydrogel components. The broad peak at 3398 cm^−1^ corresponds to O-H stretching of agarose and TA, while the peak near 1890 cm^−1^ originates from the C = O stretching of TA ester groups (Figure 2C). Decreasing agarose content weakened these peaks, likely due to the dilution effect of MP@TA and strong hydrogen-bonding interactions among MP, TA and agarose, which reduced the number of free functional groups. These spectral shifts confirm that the hydrogel is stabilized by extensive intermolecular interactions rather than simple physical mixing (Figure 2C). Rheological analysis showed that the storage modulus (*G*′) remained consistently higher than the loss modulus (*G*″) across the tested frequency range, indicating typical elastic gel behavior and good structural stability suitable for dynamic wound coverage (Figure 2D and Supplementary Figure S1A–D).

To assess the structural stability and hydration capacity of the A/MP@TA system, the mechanical properties and the water absorption capacity were evaluated (Figure 2E and Supplementary Table S2). Water absorption showed that all GHs swell rapidly in water, with wet-weight increases of approximately 10-, 11-, 13- and 20-fold over the dry weight of A/MP@TA_3_, A/MP@TA_2_, A/MP@TA_1_ and agarose, respectively (Figure 2E). It indicates that agarose has a superior water-absorption capacity, which may not make it an ideal wound dressing. The mechanical property of A/MP@TA GHs was evaluated using cyclic compression with various weights (20 g, 30 g and 50 g; Supplementary Table S2). In the wet state, all GHs can withstand 50-g weight compression (stiffer than agarose) and recover upon release of pressure. The deformation of all GHs was similar, indicating a similar stiffness across the three groups.

Compared with the highly porous and hydrophilic agarose hydrogel, the A/MP@TA GH exhibited substantially lower water uptake and SRs, indicating a denser hydrogel network. The strong supramolecular interactions among GH particles generated a compact architecture with a higher effective crosslinking density, thereby restricting polymer chain relaxation and limiting volumetric expansion upon hydration. Such restrained swelling behavior may be advantageous for wound management, as excessive water absorption can lead to overhydration of the wound bed, tissue maceration and an increased risk of bacterial infection. Moreover, the enrichment of TA-derived aromatic domains creates hydrophobic microenvironments within the hydrogel network, enabling the efficient encapsulation of hydrophobic molecules and their sustained release. The combination of limited swelling and controlled drug retention makes A/MP@TA particularly suitable for infected wound dressings, minimizing secondary tissue damage associated with hydrogel expansion while providing prolonged local delivery of bioactive agents. These structural and functional characteristics collectively contribute to a stable wound microenvironment that supports infection control and regenerative healing.

TA release profiles exhibited an initial burst followed by sustained diffusion-controlled release under both PBS and H_2_O_2_ conditions (Figure 2F and Supplementary Figure S1E–G). A similar amount of TA was released with cumulative releases of approximately 16.6%, 8.3% and 5.7% from A/MP@TA_1_, A/MP@TA_2_ and A/MP@TA_3_, respectively, after 168 h (7 days; Figure 2F and Supplementary Figure S1E–G). Since the free TAs have been washed away from GHs, we speculate that the burst release is from the outer layer of the GHs. Due to its porous internal structure, the remaining TAs are released slowly from the GH center. Interestingly, only a small percentage of TA was released while most TAs remained integrated within the particle structure, functioning as a long-term reservoir. SEM imaging confirmed that the granular morphology remained intact after one month (Figure 2F), indicating that TA participates in structural assembly and stability rather than in simple physical loading, consistent with our and others’ results [9, 19, 20]. Due to weak hydrogen bonds and a high diffusion coefficient, the released TAs could be low-molecular-weight compounds (degree of polymerization = 5–8), which are easier to release, penetrate cells more quickly and have different functions than high-molecular-weight compounds [21, 22].

The transition from a granular-dominant morphology (A/MP@TA_4-6_) to an agarose-dominated lamellar structure (A/MP@TA_1-3_) demonstrates the versatility of this design in balancing mechanical support with bioactivity. By intentionally investigating the A/MP@TA_1–3_ groups, this study establishes a ‘functional threshold’ for TA-based GHs. This approach reveals that while the agarose framework provides superior scaffolding for wound coverage, a minimum critical density of MP@TA particles is required to trigger significant therapeutic responses, such as bacterial inhibition and ROS scavenging [23]. The verification of hydrophobic microdomains via Nile Red staining highlights a key functional advantage of this granular design. The phase-separated MP@TA particles act as localized ‘hydrophobic reservoirs’ within an otherwise highly hydrophilic agarose matrix. This amphiphilic nature suggests that the GH could serve as a dual-delivery platform, capable of sequestering hydrophobic small-molecule drugs within the particles while maintaining a moist, permeable environment for oxygen and nutrient exchange through the porous agarose network.

### Biocompatibility of A/MP@TA GHs

**Figure 3:**
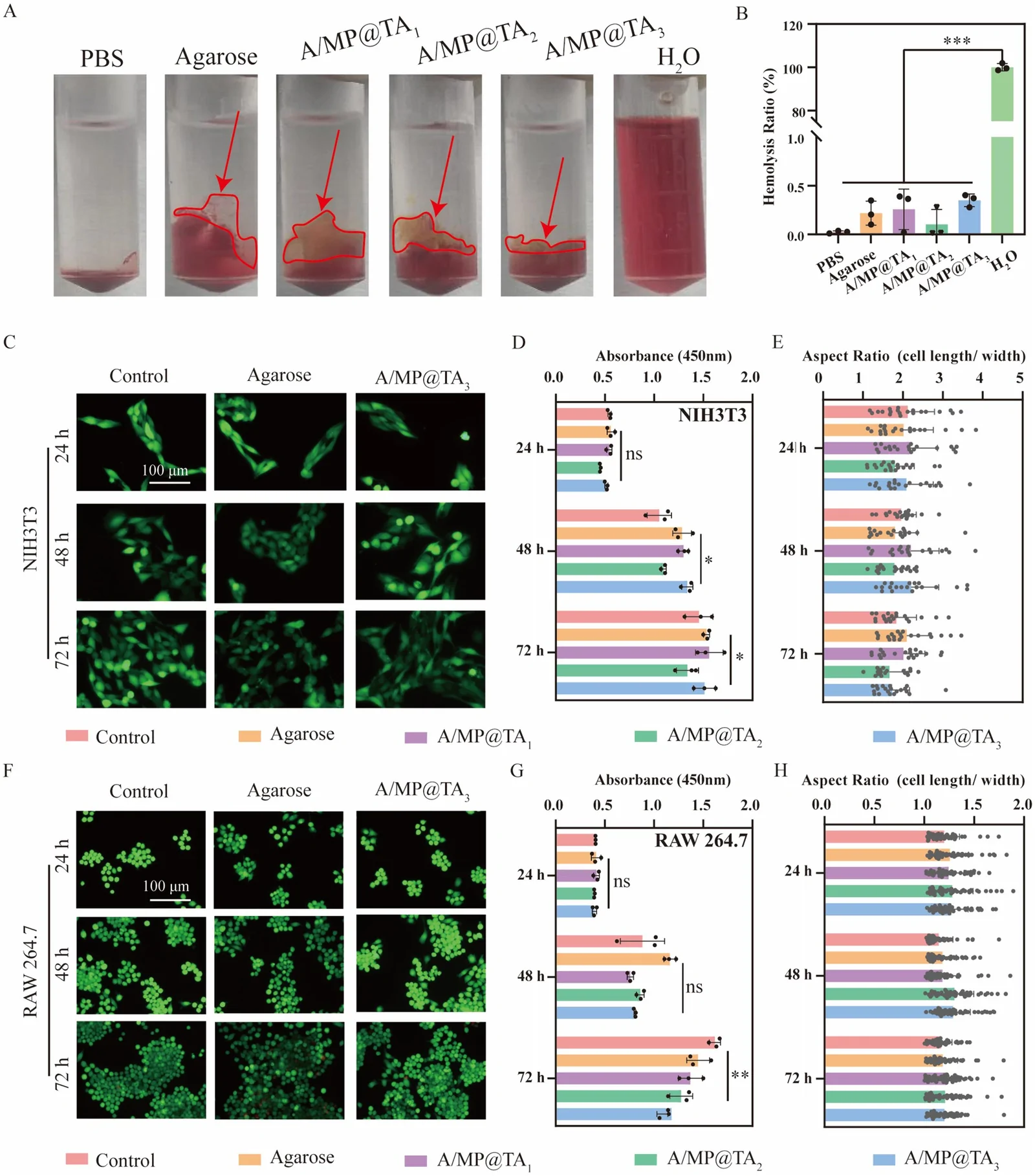
Evaluation of the hemocompatibility and cytocompatibility of A/MP@TA hydrogels. (**A**) Visual assessment of the hemolysis test. Photographs of centrifuge tubes containing PBS (negative control), H_2_O (positive control) and hydrogel samples. Arrows indicate the hydrogel specimens immersed in the PBS suspension. (**B**) Quantitative hemolysis analysis. Statistical representation of hemolysis ratios (%) for various hydrogel groups. All samples exhibit hemolysis rates significantly below the 5% safety threshold. (**C**–**E**) Cytocompatibility with NIH3T3 fibroblasts. (**C**) Representative fluorescence live/dead staining images (scale bar: 100 μm), (**D**) cell proliferation kinetics determined by CCK-8 assay (absorbance at 450 nm) over 72 h, and (**E**) the AR of NIH3T3 cells within the range of 0.8 < AR < 0.95. (**F**–**H**) Cytocompatibility with RAW 264.7 macrophages. (**F**) Live/dead staining images (scale bar: 200 μm), (**G**) quantitative cell viability via CCK-8 assay across the 72-h incubation period, and (**H**) the AR of RAW 264.7 cells within the range of 0.45 < AR < 0.75. Data are expressed as mean ± SD. Statistical significance is indicated by *P < 0.05, **P < 0.01 and ***P < 0.001.

The biocompatibility of three A/MP@TA hydrogels was evaluated to exclude cytotoxic effects from residual monomers or degradation products. Hemolysis assays demonstrated that all hydrogel samples exhibited a hemolysis effect below 2% (Figure 3A and B), well below the accepted safety threshold of 5% for biomaterials [24].

Live/dead staining of NIH3T3 fibroblasts and RAW264.7 macrophages in A/MP@TA_3_ extract revealed high cell viability (>95%) over 72 h, indicating negligible cytotoxicity and good cell compatibility (Figure 3C and F and Supplementary Figure S2A and B). CCK-8 assays confirmed that in the A/MP@TA3 extract, there was stable fibroblast proliferation (no significant difference) over 72 h (Figure 3D). On the other hand, macrophage growth in the A/MP@TA_3_ extract was slower than in the TCP control, resulting in an approximately 31.2% lower OD value on Day 3 (Figure 3G). Cell morphology analysis showed that fibroblasts exhibited normal spreading morphology (aspect ratio [AR] ∼ 2.0 and circularity ∼ 0.6, Figure 3E and Supplementary Figure S2C) over 48 h across all groups [25]. The morphology became slightly shorter (AR ∼1.6) on Day 3 due to cell-cell contacts. Macrophages maintained a rounded morphology (AR ∼1.2, circularity ∼0.9; Figure 3H and Supplementary Figure S2D) over 3 days, indicating that the cells did not polarize toward a proinflammatory M1 phenotype.

Fibroblasts and macrophages are key cellular regulators of wound repair, with their reciprocal communication coordinating inflammation, extracellular matrix remodeling, and tissue regeneration [26, 27]. Beyond direct cell–cell interactions, exosome-mediated paracrine signaling provides an important mechanism for these cells to exchange regulatory cues and coordinate the transition from inflammation to regeneration [28, 29]. In this context, A/MP@TA GH’s ability to support sustained fibroblast proliferation while maintaining macrophages in a non-inflammatory M0/M2-dominant state suggests that the hydrogel establishes a permissive microenvironment for regenerative cell–cell communication. Such coordinated fibroblast–macrophage interactions may facilitate inflammation resolution and subsequent tissue remodeling, providing a mechanistic basis for the favorable cytocompatibility and enhanced regenerative efficacy of A/MP@TA GH [30].

### Antioxidant abilities of A/MP@TA GHs

**Figure 4:**
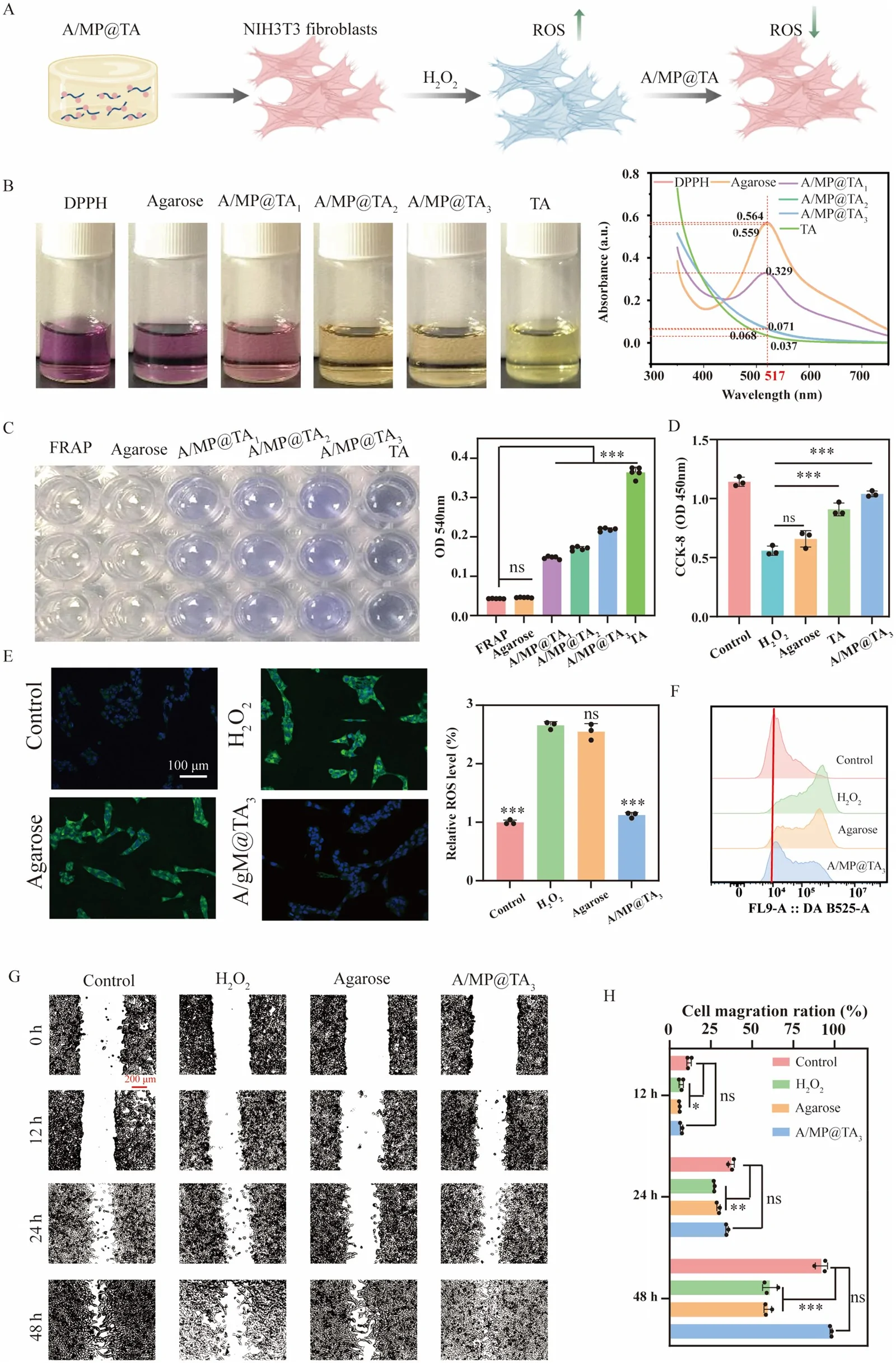
Evaluation of the antioxidant properties and *in vitro* cytoprotective effects of A/MP@TA hydrogels. (**A**) Schematic representation of the antioxidant mechanism. Illustration of A/MP@TA hydrogels scavenging H_2_O_2_-induced ROS in NIH3T3 fibroblasts, thereby restoring cellular homeostasis. (**B**) DPPH radical scavenging capacity. Visual colorimetric change and UV–Vis absorption spectra (at 517 nm) of DPPH radicals treated with different hydrogel formulations and pure TA, showing the concentration-dependent scavenging efficiency. (**C**) Ferric reducing antioxidant power (FRAP). Representative image of the FRAP assay and corresponding quantitative analysis of reducing ability based on absorbance at 540 nm. (**D**) *In vitro* cell viability. Statistical analysis of NIH3T3 cell viability (CCK-8 assay) under H_2_O_2_-induced oxidative stress, demonstrating the protective effect of the A/MP@TA_3_ hydrogel. (**E**, **F**) Intracellular ROS scavenging assessment. (**E**) Representative DCFH-DA fluorescence staining images (scale bar: 100 μm), relative intracellular ROS levels quantified via fluorescence intensity and (**F**) representative flow cytometry histograms showing the distribution of ROS-positive cells. (**G**, **H**) *In vitro* scratch wound-healing assay. (**G**) Representative optical images of NIH3T3 cell migration at 0, 12, 24 and 48 h post-wounding under oxidative stress conditions and (**H**) corresponding statistical quantification of the cell migration rate (%). Data are expressed as mean ± SD (*n* = 3). Statistical significance is indicated by *P < 0.05, **P < 0.01, ***P < 0.001 and ns (no significant).

TA plays a central role in the biological activity of A/MP@TA hydrogels (Figure 4A). DPPH radical-scavenging (hydrogen-atom transfer; Figure 4B) and FRAP assays (single-electron transfer) (Figure 4C) confirmed that the hydrogel retained strong antioxidant capacity comparable to that of free TA, with activity increasing proportionally with phenolic hydroxyl concentration. The phenolic groups in TA act as natural electron donors, neutralizing free radicals and interrupting ROS chain reactions.

In a cellular oxidative stress model, A/MP@TA hydrogels significantly protected fibroblasts from H_2_O_2_-induced oxidative damage (Figure 4D). DCFH-DA fluorescence imaging and flow cytometry further demonstrated that hydrogel extracts markedly reduced intracellular ROS levels (Figure 4E and F). This indicates that released TA not only scavenges extracellular radicals but also penetrates cellular environments to protect critical organelles such as mitochondria and DNA. Fibroblast migration assays showed that A/MP@TA treatment restored cell motility under oxidative stress conditions (Figure 4G and H). This effect may be associated with ROS elimination and the regulation of signaling pathways involved in cell migration and oxidative defense.

The biological impact of ROS on wound healing is fundamentally concentration-dependent, where both deficiency and excess impair repair [31]. At low physiological levels, ROS act as essential secondary messengers that activate key signaling cascades, including the PI3K/Akt, MAPK and NF-κB pathways, which are critical for triggering cell proliferation and migration [32, 33]. However, persistent high ROS concentrations in the wound microenvironment lead to chronic oxidative stress, which induces mitochondrial dysfunction and DNA damage, ultimately stalling the healing process [34]. Specifically, excessive ROS levels inhibit fibroblast migration by disrupting cytoskeletal dynamics and promoting a pro-fibrotic environment, leading to hypertrophic scarring [35]. The A/MP@TA GH addresses this by modulating redox balance and scavenging excess radicals; the hydrogel prevents overactivation of inflammatory pathways while potentially supporting anti-inflammatory pathways, such as the Nrf2 pathway, which orchestrates the cellular antioxidant defense system [31, 33]. Restoring fibroblast motility under these conditions is vital, as timely cell recruitment and balanced collagen deposition are the primary determinants for functional tissue regeneration and the prevention of excessive fibrotic scarring [33, 35]. Therefore, the antioxidant capacity of A/MP@TA GH not only eliminates ROS but also restores a signaling-friendly environment that favors regenerative migration over pathological fibrosis.

### Antibacterial properties of A/MP@TA GHs

**Figure 5:**
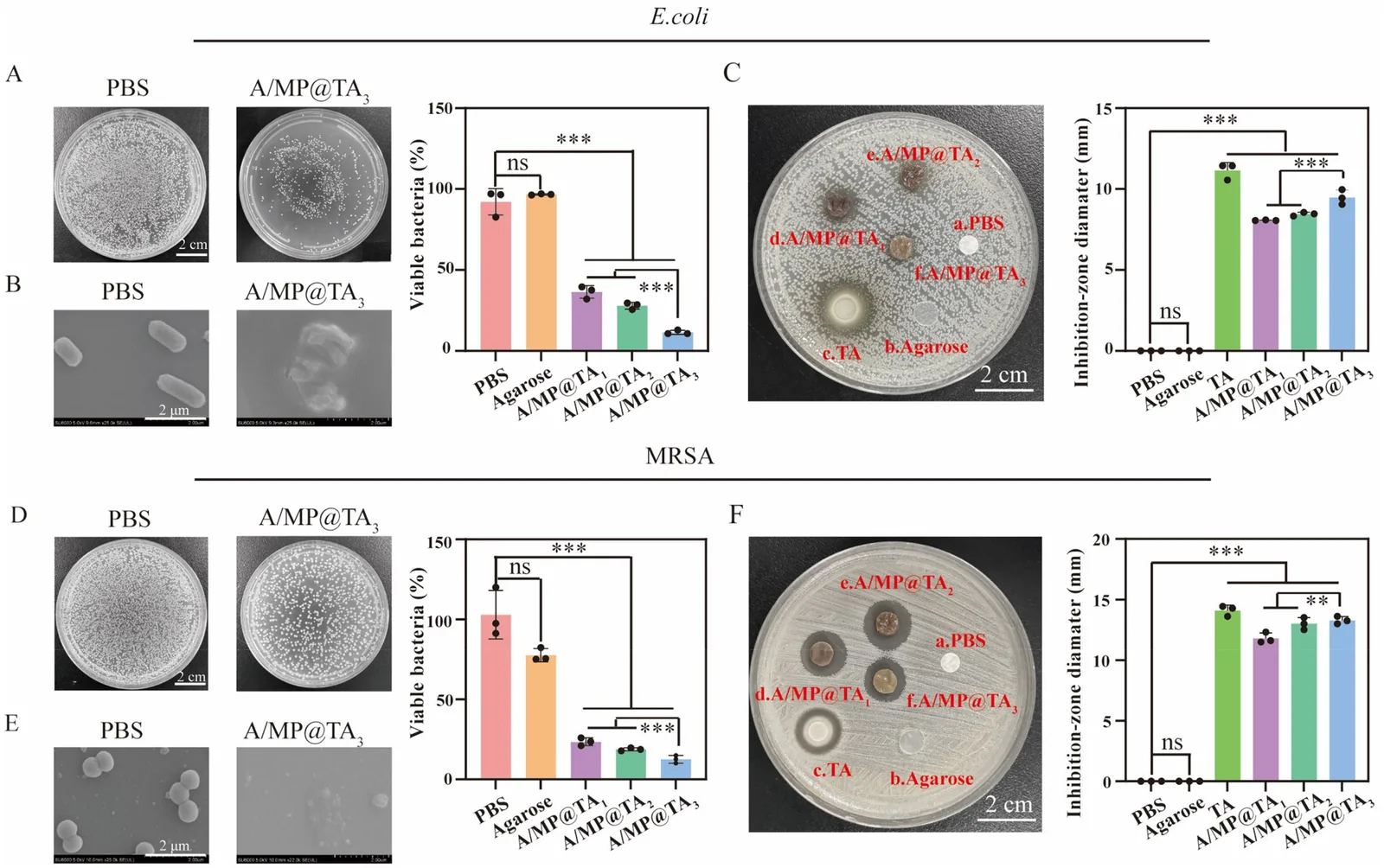
*In vitro* antibacterial performance of A/MP@TA hydrogels against Gram-negative and Gram-positive bacteria. (**A**–**C**) Antibacterial activity against Escherichia coli (E. coli). (**A**) Representative photographs of agar plates showing colony formation and corresponding quantitative analysis of viable bacteria (%) after treatment with PBS and A/MP@TA_3_ hydrogel formulations. (**B**) SEM images of *E. coli* under different treatments. (**C**) Agar disk diffusion assay for *E. coli*. Visual observation of inhibition zones (a: PBS; b: Agarose; c: TA; d–f: A/MP@TA_1-3_) and statistical quantification of the inhibition zone diameters (mm). (**D**–**F**) Antibacterial activity against MRSA. (**D**) Colony formation images on agar plates and statistical quantification of bacterial viability (%) demonstrating the potent inhibitory effect of the hydrogels on MRSA. (**E**) SEM images of MRSA under different treatments. (**F**) Agar disk diffusion assay for MRSA. Representative images of inhibition zones and corresponding quantitative diameters (mm) for the various experimental groups. Data are presented as mean ± SD (*n* = 3). Statistical significance is indicated by *P < 0.05, **P < 0.01, ***P < 0.001, while ‘ns’ indicates no significant difference.

Effective antibacterial activity is essential for advanced wound dressings. A/MP@TA hydrogels were evaluated against *E. coli* and MRSA using colony counting and inhibition zone assays (Figure 5). *Staphylococcus aureus* (SA) is a major pathogen in skin infections, while *E. coli* commonly contaminates traumatic wounds.

The antibacterial activity of GH was analyzed using a colony-forming unit (CFU) assay and a direct bactericidal effect assay (inhibition zone assay). The CFU assay showed that three GHs kill more than 60% of *E. coli*, especially A/MP@TA_3_, which kills 90% of bacteria, and the membrane of *E. coli* was disrupted (Figure 5A and B and Supplementary Figure S3A and B). Three GHs had approximately 8-mm-diameter inhibition zone of *E. coli* (Figure 5C). At the same time, three GHs killed more than 70% of MRSA and the membrane of MRSA was also disrupted under A/MP@TA_3_ treatment (Figure 5D and E and Supplementary Figure S3C and D). Three GHs had approximately 12-mm-diameter inhibition zone of MRSA (Figure 5F). The ability of A/MP@TA hydrogels to inhibit both bacteria indicates broad-spectrum antibacterial activity, making them suitable for complex, infected wound environments.

Unlike conventional antibiotics, which typically target specific biochemical pathways, TA exerts a broad-spectrum antimicrobial effect through a multitarget approach that minimizes the development of bacterial resistance [36]. This multifaceted mechanism primarily involves the physical disruption of bacterial cell walls and membranes through strong hydrophobic interactions and hydrogen bonding, leading to the leakage of intracellular components and eventual lysis [37]. Additionally, TA can irreversibly form complexes with bacterial surface proteins and metabolic enzymes, effectively neutralizing their activity and causing metabolic collapse [38]. The high affinity of TA for essential metal ions also starves bacteria of necessary nutrients, further enhancing its inhibitory effect on both Gram-positive and Gram-negative strains [37]. Because TA acts through these nonspecific and synergistic pathways, it maintains potent efficacy against multidrug-resistant pathogens, such as MRSA, without triggering the selective pressure commonly associated with single-site antibiotics [39]. Moreover, the granular microstructure increases the effective contact area and promotes sustained TA release, enabling prolonged antibacterial efficacy.

### Pathways involved in A/MP@TA GH-mediated macrophage polarization

**Figure 6:**
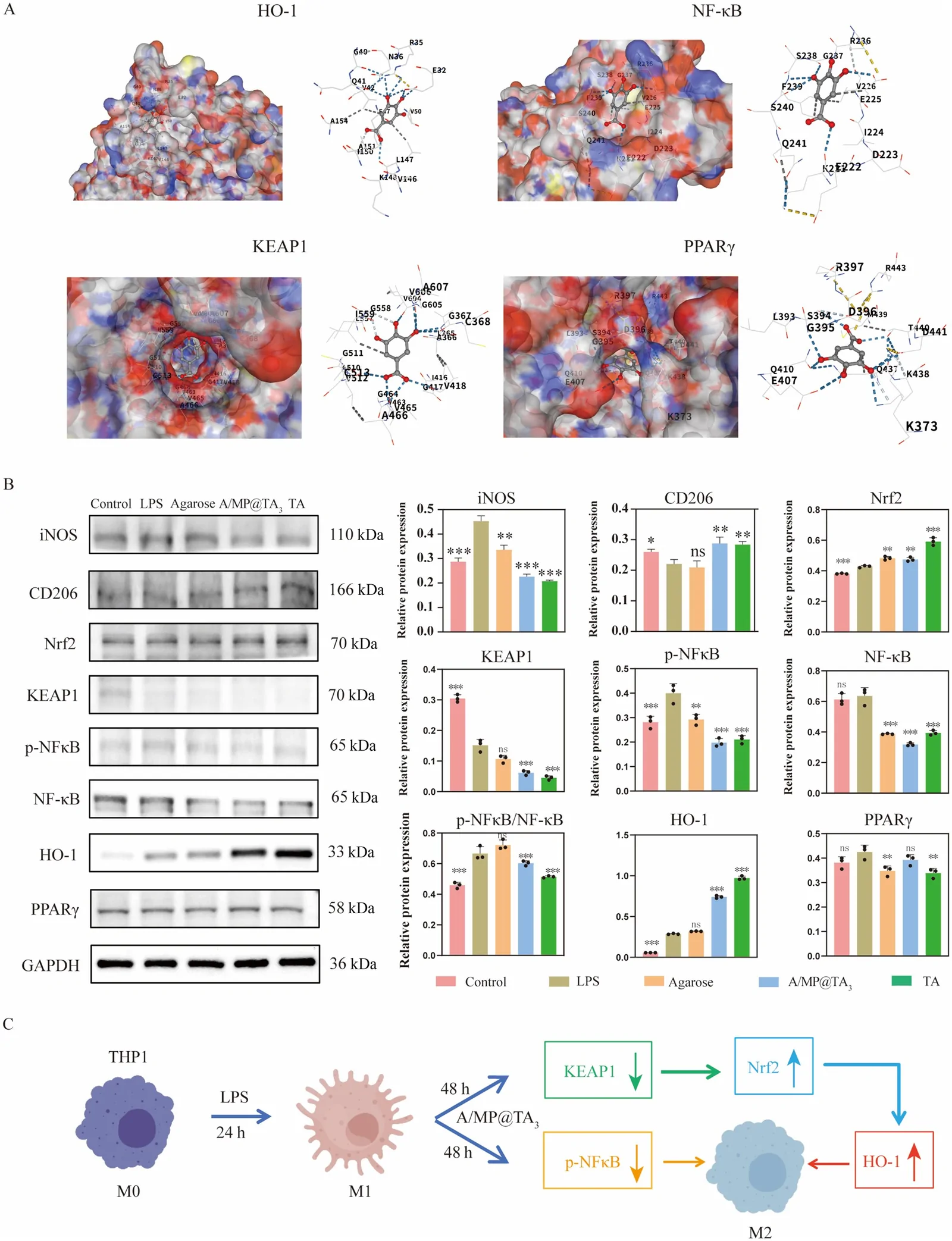
Analysis of hydrogel-activated targets and downstream signaling pathways of THP-1 macrophages. (**A**) Molecular docking analysis of gallic acid (CID 370) with HO-1, Keap1, NF-κB, and PPARγ. (**B**) Western blot analysis of iNOS, CD206, Nrf2, Keap1, p-NFκB, total NF-κB, HO-1 and PPARγ. (**C**) Schematic representation of potential TA-regulated THP‑1 macrophages. Data are presented as mean ± SD (*n* = 3). Statistical significance is indicated by *P < 0.05, **P < 0.01 and ***P < 0.001.

Given the critical role of the immune microenvironment in orchestrating tissue regeneration, we next sought to identify the molecular targets underlying TA’s immunomodulatory activity. The effects of TA-loaded A/MP@TA_3_ GH on LPS-challenged THP-1 macrophages were therefore investigated. Blind docking analysis using CB-Dock2 revealed that gallic acid (CID: 370), the principal phenolic motif of TA, exhibited favorable interactions with several key regulators of macrophage polarization and inflammatory signaling, including HO-1 (PDB: 3HOK), NF-κB p65 (PDB: 1BFT), KEAP1 (PDB: 4L7B) and PPARγ (PDB: 2PRG). Among these targets, KEAP1 and PPARγ displayed the strongest predicted binding affinities, with Vina scores of −6.9 and −6.5 kcal/mol, respectively, suggesting a potential involvement of the KEAP1/Nrf2 pathways in TA-mediated immunoregulation (Figure 6A and Supplementary Figure S4).

We further investigated the regulatory effects of A/MP@TA_3_ GH on pro-inflammatory M1 macrophages. M0 macrophages were first polarized into M1 inflammatory macrophages by LPS stimulation for 24 h, followed by treatment with A/MP@TA_3_ GH for an additional 48 h for protein expression analysis. The results showed that treatment with A/MP@TA_3_ GH significantly upregulated the expression of Nrf2, HO-1 and CD206, while suppressing the levels of KEAP1, iNOS and phosphorylated NF-κB (p-p65) in M1 macrophages (Figure 6B).

Our integrated computational and experimental analyses reveal a previously underappreciated hierarchical signaling network through which TA coordinates macrophage polarization. Blind docking predicts that gallic acid, the core pharmacophore of TA, directly engages PPARγ, KEAP1 and NF-κB (p65), implying that TA acts as a multitarget immunomodulator. At the top of the regulatory hierarchy, PPARγ emerges as a master upstream node that can simultaneously transactivate Nrf2 gene expression and promote the proteasomal degradation of the p65 subunit of NF-κB [40, 41]. However, PPARγ expression was similar across groups, indicating that the released TA did not alter total PPARγ expression but rather acted via direct ligand binding, phosphorylation-mediated activation and exchange of ligand-dependent co-regulatory factors.

On the other hand, consistent with this architecture, TA treatment resulted in marked downregulation of KEAP1, the principal cytosolic repressor of Nrf2, leading to Nrf2 accumulation. Liberated Nrf2 subsequently suppressed the phosphorylation of p65 (Ser536), as evidenced by reduced p-p65 levels without a commensurate decrease in total p65 expression [42–44]. This selective attenuation of p65 activity abrogated the transcriptional output of the canonical NF-κB pathway, as reflected by diminished iNOS (a classic M1 hallmark) and reciprocal elevation of CD206 (an M2-associated marker) [4, 45].

Moreover, the data support a bidirectional crosstalk between the KEAP1/Nrf2 and NF-κB pathways. While Nrf2 represses NF-κB activation by limiting oxidative stress and interfering with IKK complex assembly, p65 can, in turn, promote KEAP1 nuclear translocation, thereby facilitating Nrf2 degradation [46–48]. The TA-induced suppression of KEAP1 likely disrupts this feedback loop, tilting the equilibrium toward sustained Nrf2 activity and durable NF-κB inhibition. Collectively, our findings position TA as a hierarchical pathway modulator that integrates PPARγ-driven transcriptional priming, KEAP1/Nrf2-mediated redox sensing and NF-κB-dependent inflammatory execution, ultimately steering macrophages from a pro-inflammatory M1 toward an anti-inflammatory M2 phenotype.

### *In vivo* infected wound healing assay

**Figure 7:**
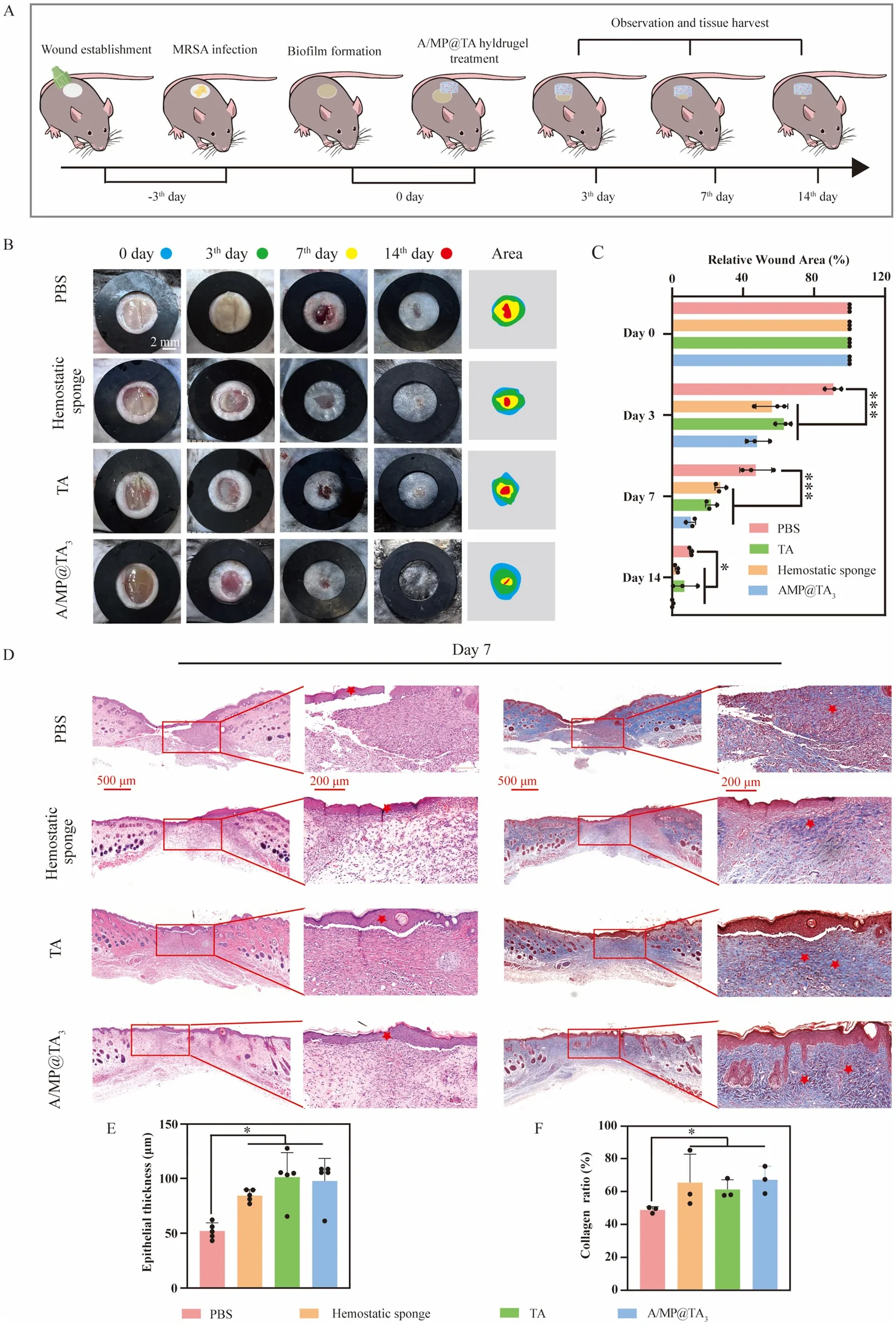
*In vivo* therapeutic efficacy of A/MP@TA hydrogels in an MRSA-infected skin wound model. (**A**) Experimental timeline and procedure. Schematic workflow of the animal study, including the establishment of the full-thickness wound, MRSA infection, biofilm formation and subsequent treatment with various groups over a 14-day observation period. (**B**) Macroscopic wound healing progress. Representative digital photographs and corresponding digitized area traces of infected wounds treated with PBS, commercial hemostatic sponge, pure TA and A/MP@TA_3_ hydrogel at 0, 3, 7 and 14 days post-treatment. (**C**) Quantitative wound closure kinetics. Statistical analysis of the relative wound area (%) over time (at Days 0, 3, 7 and 14 post-treatment), illustrating the accelerated healing rate in the A/MP@TA_3_ group. (**D**) Histological evaluation of wound regeneration. Representative Hematoxylin and Eosin (H&E)-stained (left) and Masson’s trichrome-stained (right) tissue sections from the wound sites at Day 7. Magnified insets provide detailed views of the re-epithelialization and collagen deposition (scale bars: 200 μm). (**E**, **F**) Quantitative histological analysis. (**E**) Morphometric quantification of the mean epithelial height based on H&E staining and (**F**) statistical analysis of the collagen area ratio (%) derived from Masson’s trichrome staining, reflecting the quality of dermal remodeling. Data are presented as mean ± SD (*n* = 3). Statistical significance is indicated by *P < 0.05, **P < 0.01 and ***P < 0.001.

The therapeutic performance of A/MP@TA GH was further evaluated in an MRSA-infected full-thickness skin wound model in C57BL/6 mice (Figure 7A). Treatment began 3 days after infection to simulate established biofilm conditions. Histocompatibility results indicate that the hydrogel has no toxic effects on the internal organs (heart, liver, spleen, lungs, and kidneys) of the mice (Supplementary Figure S5). A/MP@TA_3_ significantly accelerated wound closure at Days 3 and 7 compared with the commercial absorbable gelatin hemostatic sponge and the TA solution. By Day 14, wounds treated with A/MP@TA_3_ were almost completely healed (Figure 7B and C).

Histological analysis revealed more complete re-epithelialization and organized dermal structures in the A/MP@TA group (Figure 7D). Notably, increased neovascularization and hair follicle regeneration were observed, indicating high-quality regenerative healing rather than simple scar formation (Figure 7E). Masson staining further revealed dense, well-aligned collagen fibers, suggesting improved ECM remodeling (Figure 7F).

The enhanced healing effect arises from the combined actions of antibacterial activity, ROS scavenging, immunomodulation and the hydrogel network’s moist microenvironment. Previous studies have emphasized that the synchronized orchestration of neovascularization and ECM remodeling is a prerequisite for achieving functional, scar-free tissue recovery [14]. The early establishment of a robust vascular network is a critical driver of regenerative healing, as it ensures the efficient delivery of nutrients and regulatory signaling molecules that foster a benign healing cycle [14]. This positive feedback loop accelerates the transition from the inflammatory phase to the remodeling stage, thereby minimizing the duration of fibrotic signaling that typically leads to scar formation [14]. Furthermore, the synthesis of well-organized collagen fibers and the balanced ratio of collagen subtypes are essential indicators of regenerative quality, distinguishing true skin restoration from simple fibrotic repair [14]. By providing a biomimetic microenvironment, the A/MP@TA GH likely promotes these regenerative pathways, ensuring that the newly formed tissue mirrors the structure of healthy skin and can potentially prevent hypertrophic scar formation.

### Immunomodulation and tissue regeneration

**Figure 8:**
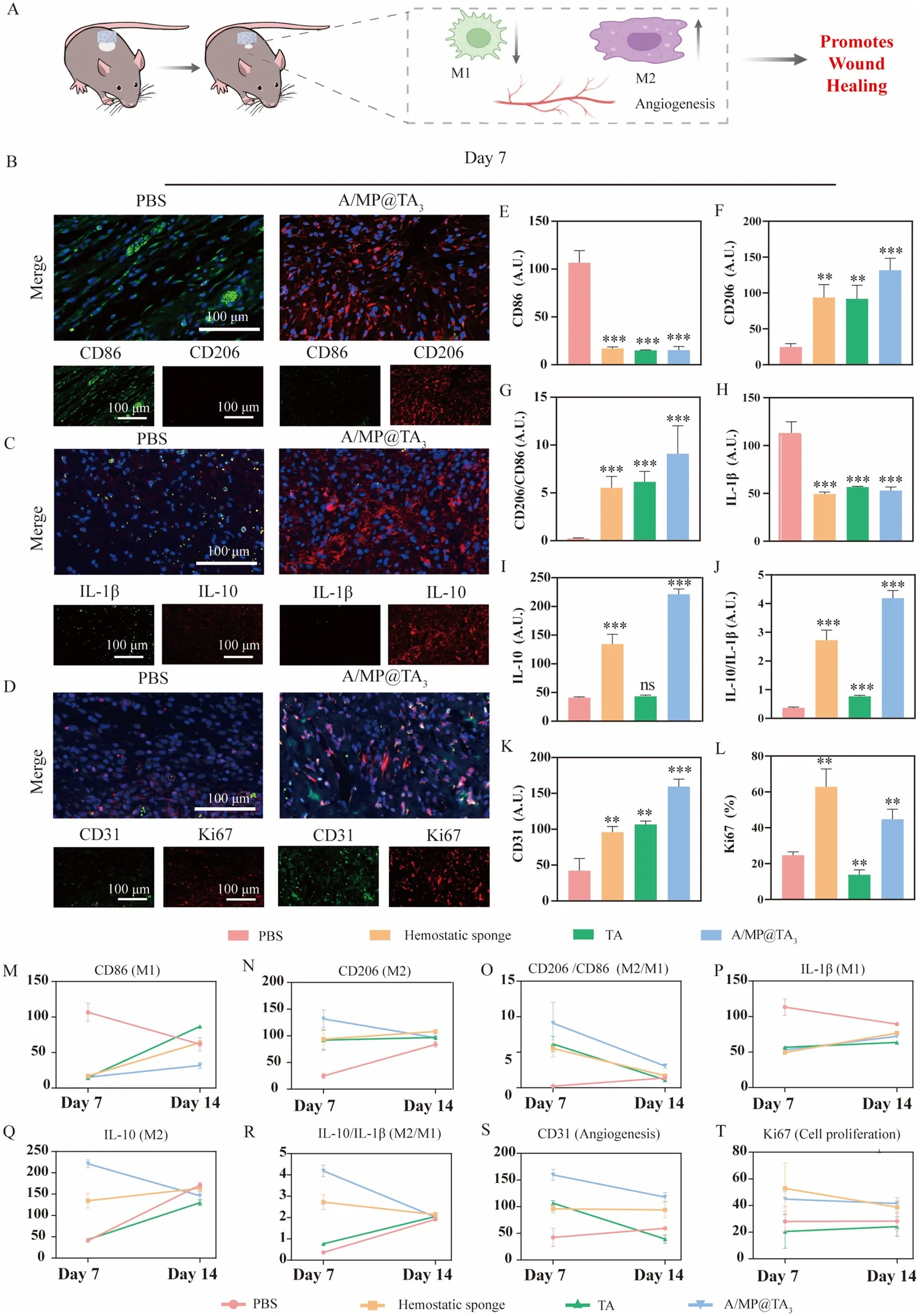
Spatiotemporal analysis of immunomodulatory and pro-regenerative functions of A/MP@TA GH in infected wounds. (**A**) Schematic illustration showing the hydrogel’s role in modulating macrophage polarization (promoting the M1-to-M2 transition), stimulating angiogenesis and accelerating the overall wound healing process. (**B**–**D**) Representative immunofluorescence images. Staining of wound tissue sections on Day 7 for (**B**) M1 macrophage marker CD86 (green) and M2 macrophage marker CD206 (red), (**C**) M1 macrophage marker IL-1β (green) and M2 macrophage marker IL-10 (red) and (**D**) angiogenic marker CD31 (green) and cell proliferation marker Ki67 (red). Nuclei were counterstained with DAPI (blue). (Scale bars: 100 μm). (**E**–**L**) Quantitative expression analysis at Day 7. Mean fluorescence intensity (MFI) Quantification of (**E**) CD86, (**F**) CD206, (**H**) IL-1β, (**I**) IL-10, (**K**) CD31 and (**L**) Ki67, along with (**G**) the CD206/CD86 ratio and (**J**) the IL-10/IL-1β ratio, indicates an anti-inflammatory effect and the microenvironment shifts toward promoting pro-healing. (**M**–**T**) Time-dependent changes in the expression of (**M**) CD86, (**N**) CD206, (**O**) CD206/CD86, (**P**) IL-1β, (**Q**) IL-10, (**R**) IL-10/IL-1β, (**S**) CD31 and (**T**) Ki67 across the different treatment groups from Day 7 to Day 14, highlighting the sustained regenerative effect of the A/MP@TA_3_ hydrogel. Data are presented as mean ± SD (*n* = 3). Statistical significance is indicated by *P < 0.05, **P < 0.01 and ***P < 0.001.

The ratio of macrophage subtypes (M2/M1 cells) at the wound site is vital during wound healing (Figure 8A). Immunofluorescence analysis revealed increased M2 macrophages (upregulation of CD206 and IL-10 and downregulation of CD86 and IL-1β) and elevated CD31 (endothelial cells) and VEGF expression in the A/MP@TA_3_ group at Day 7 (Figure 8B–D and Supplementary Figure S6). Newly formed blood vessels can provide oxygen and nutrients to support granulation tissue growth and long-term tissue remodeling. At Day 14, M1 macrophages remained suppressed compared with other treatments, indicating accelerated immune balance (Supplementary Figure S7A–C, S8C and S9A–D).

In a normal wound-healing microenvironment, recruitment of M1 macrophages is required during the initial inflammatory stage, followed by M2-mediated tissue repair. A/MP@TA_3_ treatment significantly reduced CD86 and IL-1β expression and increased CD206 and IL-10 expression, thereby demonstrating microenvironmental immune balance. Interestingly, at week 1, no significant difference in M1 macrophage levels was observed between the A/MP@TA_3_ group and the other groups, whereas the proportion of M2 macrophages was relatively higher. By week 2, however, the A/MP@TA_3_ group exhibited significantly reduced M1 macrophage levels, while the proportion of M2 macrophages remained comparable to that in the other groups (Figure 8M–R and Supplementary Figure S8A and B). These findings suggest that the GH treatment may rapidly activate the early immune response at the wound site and facilitate the timely resolution of the pro-inflammatory phase, leading to a rapid decline of M1 macrophages. Notably, this effect does not rely on recruiting a substantially larger population of M2 macrophages than the other groups; rather, it appears to promote a more efficient transition of the immune microenvironment toward a regenerative state, thereby supporting gradual wound repair.

Persistent CD31 upregulation and Ki67 expression indicated enhanced angiogenesis and cellular proliferation at Day 7 (Figure 8K and L and Supplementary Figure S8J). CD31 expression around the wound site in the A/MP@TA group remained consistently high from Day 7 to Day 14. In contrast, CD31 levels in the other groups exhibited divergent trends, gradually decreasing in the control and hemostatic sponge groups while showing only a modest increase in the TA-treated group during the same period (Figure 8S). Cell proliferation in the A/MP@TA_3_ group remained strongly activated from Day 7 to Day 14, whereas the TA group exhibited only a gradual increase over the same period (Figure 8T and Supplementary Figure S8K). In combination with macrophage phenotype analysis, this pattern indicates that proliferating cells in the A/MP@TA group were primarily associated with a reparative M2 microenvironment, whereas the other groups largely remained in an immune-stress state.

Previous studies have demonstrated that the timely transition of the immune microenvironment, characterized by the polarization of macrophages from a pro-inflammatory M1 phenotype to a pro-regenerative M2 phenotype, is a prerequisite for orchestrating functional tissue repair [15]. In our system, this phenotypic switch is closely associated with the recruitment of CD31-positive endothelial cells and sustained VEGF release, which promotes angiogenesis to meet the high metabolic demands of the regenerating dermis (Supplementary Figure S9). This coordinated immune-vascular response not only accelerates wound closure but also limits excessive collagen deposition, thereby reducing the risk of hypertrophic scarring and improving the quality of skin regeneration [49]. The synergistic interplay between anti-inflammatory signaling and VEGF-mediated neovascularization not only accelerates wound closure but also enhances the structural integrity and functional recovery of the skin [50]. Furthermore, a well-regulated immune-vascular axis prevents the prolonged inflammatory stimulus that typically drives excessive collagen deposition, thereby playing a decisive role in mitigating the risk of hypertrophic scarring and promoting aesthetic regenerative outcomes [51]. By synergistically modulating the macrophage-driven immune microenvironment and ensuring the sustained bioavailability of CD31, the A/MP@TA GH facilitates a microenvironment conducive to functional skin regeneration rather than disorganized fibrotic scarring.

## Conclusion

In summary, we previously established a fully aqueous strategy for the fabrication of granular hydrogels (GHs). In this study, we further investigated the formation of a four-component GH system (A/MP@TA), providing deeper insight into the polymer assembly mechanism and functional potential of this platform. After introducing an additional polysaccharide macromolecule, the granular architecture of the MP@TA system remained stable, while its antibacterial and antioxidant activities were well preserved. These effects can be attributed to the hierarchical granular structure and the sustained release of TA. Importantly, by exploring the compositional boundaries and concentration limits required for GH formation, we demonstrated that the GH system can maintain its intrinsic biological and immunomodulatory functions even with minimal TA incorporation. In an MRSA-infected wound model, the A/MP@TA GH effectively disrupted the pathological cycle of persistent infection, inflammation and oxidative stress, thereby establishing a favorable regenerative microenvironment that accelerates wound regeneration. Overall, this study provides new insights into the design principles of polyphenol-mediated GH systems and offers a green, versatile strategy for constructing multifunctional biomaterials for the treatment of infected wounds.

## Supplementary Material

rbag158_Supplementary_Data
