# Supplementary material for: Bioactive granular hydrogels for infection control and immune microenvironment remodeling in wound regeneration
Source: Regen Biomater. 2026 Jul 29;13:rbag158. doi: 10.1093/rb/rbag158 (PMC13534143; doi:10.1093/rb/rbag158)
Supplement: Supplementary file 1 [file rbag158_supplementary_data.docx]

**Supporting Information**

**Bioactive Granular Hydrogels for Infection Control and Immune Microenvironment Remodeling in Wound Regeneration**

Yuyan Hua^1,2^, Yung-Chiang Liu^1,2^, Zhili Qiu^1,2^, Yin Liu^1^, Guoyan Li^1^, Zhilin Wei^1^, Yuefang Shen^1^, Huiyuan Chen^1^, Bihui Ren^1^, Enhui Zhou^1^, Jinfeng Wang^1^, Duoduo Zhang^1^, Peng-Yuan Wang^1,^*

1. Oujiang Laboratory; Key Laboratory of Alzheimer’s Disease of Zhejiang Province, Institute of Aging, Wenzhou Medical University, Wenzhou, Zhejiang 325000, People’s Republic of China

2. These authors contributed equally

^*^ Corresponding author: py.wang@ojlab.ac.cn (P.-Y. Wang)

**Figure captions**

**Figure S1**. **Frequency-dependent rheological profiles and in vitro cumulative release profile of various hydrogel formulations.** (A–D) Dynamic frequency sweep tests. Storage modulus (*G'*, black symbols) and loss modulus (*G''*, red symbols) measured as a function of angular frequency (0.1 to 100 rad/s) for (A) pure agarose, (B) A/MP@TA₁, (C) A/MP@TA₂, and (D) A/MP@TA₃. In all groups, *G'* remains significantly higher than *G''* throughout the tested range, indicating the formation of a stable viscoelastic solid network. The decrease in both moduli with increasing MP/TA content suggests a modulation of the network crosslinking density. (E) Sustained release behavior of TA from the Agarose, A/MP@TA_1_, A/MP@TA_2_, and A/MP@TA₃ in PBS. (F) Sustained release behavior of TA from the Agarose, A/MP@TA_1_, A/MP@TA_2_, and A/MP@TA₃ in H_2_O_2_. (G) Sustained release behavior of TA from the Agarose, A/MP@TA_1_, A/MP@TA_2_, and A/MP@TA₃ in H_2_O_2_. The hydrogel system was monitored over 168 hours (7 days).

**Figure S2**. **Representative fluorescence live/dead staining images** of (A) NIH3T3 and (B) RAW264.7 cells. Scale bar: 200 µm. The distribution of the circularity for (C) RAW264.7 and (D) NIH3T3 cells within the range of 0.45 < Circ < 0.75. Data are expressed as mean ± SD. Statistical significance is indicated by **p* < 0.05, ***p* < 0.01, and ****p* < 0.001.

**Figure S3.** **Representative photographs of bacteria.** (A) *E.coli* colony formation. (B) *E.coli* SEM image. (C) MRSA colony formation. (D) MRSA SEM image.

**Figure S4. Molecular docking analysis.** Prediction of the potential binding sites, binding modes, and binding affinities between gallic acid (<CID:370>) and Keap1, PPARγ, HO-1, and NF-κB.

**Figure S5. Systemic biosafety evaluation of A/MP@TA hydrogels in vivo.** Representative H&E-stained histological sections of major organs, including the heart, liver, spleen, lungs, and kidneys, harvested from mice on (A) Day 7 and (B) Day 14 post-treatment. Four experimental groups, PBS, commercial hemostatic sponge, pure TA, and A/MP@TA₃ hydrogel, were evaluated. No significant histopathological abnormalities, inflammatory cell infiltration, or organ damage were observed in any group, indicating the excellent systemic biocompatibility and safety of the A/MP@TA₃ hydrogel. (Scale bars: 200 µm).

**Figure S6. Immunostaining of macrophages and endothelial cells in the wound tissues** (Day 7). The PBS control and A/MP@TA are in Figure 8.

**Figure S7. Histological analysis of wound tissues** (Day 14). (A) Dermal remodeling and collagen maturation. Representative H&E-stained (left) and Masson’s trichrome-stained (right) images of wound sections on day 14 post-treatment. Magnified views highlight the degree of re- epithelialization and the organization of the collagen matrix (Scale bars: 500 µm). (B, C) Morphometric quantification of wound regeneration. Statistical analysis of (B) the mean epithelial height and (C) the collagen area ratio (%), reflecting the final structural recovery of the skin across the experimental groups. The early-stage analysis is in Figure 7.

**Figure S8.** **Immunostaining of macrophages and endothelial cells in the wound tissues** (Day 14). (A-C) Representative immunofluorescence images of markers at Day 14. Staining of tissue sections for (A) CD86 (green) and CD206 (red), (B) IL-1β (green) and IL-10 (red) to assess macrophage persistence, and (C) CD31 (green) and Ki67 (red) to evaluate late-stage vascularization and cellular activity. Nuclei were counterstained with DAPI (blue). (Scale bars: 100 µm). (D-K) Quantitative expression analysis at Day 14. Mean fluorescence intensity quantification of (D) CD86, (E) CD206, (G) IL-1β, (H) IL-10, (J) CD31, and (K) Ki67 markers, along with (F) the CD206/CD86 ratio and (I) the IL-10/ IL-1β ratio, indicating the resolution of inflammation and the transition to the remodeling phase. Data are presented as mean ± SD (n = 3). Statistical significance is indicated by **p* < 0.05, ***p* < 0.01, ****p* < 0.001, and ns (no significant). The early-stage analysis is in Figure 8.

**Figure S9. Immunostaining of VEGF (vascular endothelial growth factor) in the wound tissues** (Days 7 and 14). (A-B) Representative immunofluorescence images of markers at Days 7 and 14. Staining of tissue sections for (A) VEGF (Day 7) and (B) VEGF (Day 14) was performed to assess macrophage-associated angiogenic activity. Nuclei were counterstained with DAPI (blue). (Scale bars: 100 µm).

**Table S1. Gelation time of five different types of hydrogels.** MP is pre-polymer; MP@TA is mixing MP and TA; MP@TA+PEGDA is mixing MP, TA, and PEGDA; A/MP@TA+PEGDA is mixing agarose, MP, TA, and PEGDA. Gelation was tested for 60 min.

**Table S2. Mechanical properties of hydrogel using static compression.** Three types of A/MP@TA GHs were compressed using different weights (20-50 g).

**Figure S1**

**Figure S2**

**Figure S3**

**Figure S4**

**Figure S5**

**Figure S6**

**Figure S7**

**Figure S8**

**Figure S9**

**Table S1. Gelation time of five different types of hydrogels.**

| Type  Time | MP  -PEGDE | MP@TA  -PEGDE | MP  +PEGDE | MP@TA  +PEGDE | A/MP@TA  +PEGDE |
| --- | --- | --- | --- | --- | --- |
| 10 min |  |  |  |  |  |
| 20 min |  |  |  |  |  |
| 30 min |  |  |  |  |  |
| 40 min |  |  |  |  |  |
| 50 min |  |  |  |  |  |
| 60 min |  |  |  |  |  |

| Type  Time | MP  -PEGDE | MP@TA  -PEGDE | MP  +PEGDE | MP@TA  +PEGDE | A/MP@TA  +PEGDE |
| --- | --- | --- | --- | --- | --- |
| 10 min | X | X | O | X | O |
| 20 min | X | X | O | X | O |
| 30 min | X | X | O | O | O |
| 40 min | X | X | O | O | O |
| 50 min | X | X | O | O | O |
| 60 min | X | X | O | O | O |

**Table S2. Mechanical properties of hydrogel using static compression.**

| Type | | A/MP@TA_1_ | A/MP@TA_2_ | A/MP@TA_3_ |
| --- | --- | --- | --- | --- |
| Original | |  |  |  |
| Compression | 20 g |  |  |  |
|  | 30 g |  |  |  |
|  | 50 g |  |  |  |
| Recovery | |  |  |  |
